# Hepatitis B and human immunodeficiency virus co-infection among pregnant women in resource-limited high endemic setting, Addis Ababa, Ethiopia: implications for prevention and control measures

**DOI:** 10.1186/s40001-016-0211-3

**Published:** 2016-04-14

**Authors:** Zelalem Desalegn, Liya Wassie, Habtamu Bedimo Beyene, Adane Mihret, Yehenew A. Ebstie

**Affiliations:** Department of Microbiology, Immunology and Parasitology, School of Medicine, Addis Ababa University, P. O. Box: 9086, Addis Ababa, Ethiopia; Armauer Hansen Research Institute (AHRI), Jimma Road, ALERT Campus, Addis Ababa, Ethiopia

**Keywords:** Hepatitis, Pregnancy, Ethiopia, Seroprevalence

## Abstract

**Background:**

Hepatitis, a highly contagious viral infection, is one of the leading killer diseases globally caused by hepatitis virus. Among the existing viral causes for hepatic failure, hepatitis B virus (HBV) plays a significant role with devastating implications, especially when combined with other viral infections such as human immunodeficiency virus (HIV). Co-infection with hepatitis B virus and HIV leads to increased morbidity and mortality as compared to independent HIV and HBV infections. In this study, we aimed to assess the seroprevalence of HBV and HIV coinfection and associated risk factors among pregnant women in a selected hospital facility around Addis Ababa, Ethiopia.

**Methods:**

A total of 215 pregnant women were recruited between July and October 2014 from Tirunesh Beijing General Hospital. A pretested and structured questionnaire was used to collect socio-demographic characteristics and possible risk factors. In addition, 5 ml venous blood was collected and centrifuged to estimate the seroprevalence of HBV and HIV. Descriptive statistics and logistic regression analysis were done and a *P* value less than 0.05 was considered statistically significant.

**Results:**

The overall prevalence of hepatitis B virus infection was 13 (6 %). This positivity was different across different age categories: 1 (11.1 %), 3 (4.5 %), 6 (6 %), 1 (3.2 %), and 2 (25 %) among those between 15–19, 20–24, 25–29, 30–34, and 35–39 years, respectively. However, a statistically significant association was not established between age and HBV. Among the total, 9 (4.2 %) of the positive cases were detected among primary school completed. Multivariate analyses indicated that history of abortion (*p* = 0.003), history of surgery (*p* = 0.0.022), and tattooing (*p* = 0.033) were significantly associated with HBV infection. A total of 9 (4.2 %) women were found to be HIV seropositive, of whom 2 (22.2 %) were co-infected with HBV.

**Conclusions:**

We observed a relatively higher seroprevalence of HBV infection among pregnant women in the study area, in which majority of the cases had underlying risk factors for acquiring the infection. Since none of the mothers were vaccinated for HBV, the possibility of perinatal transmission is inevitable. Hence, routine screening and immunization against HBV during pregnancy and health education are highly warranted to alleviate the situation.

## Background

Hepatitis B virus (HBV)-associated infection is one of the leading causes of liver diseases causing serious public health problem worldwide. Since the majority of infected individuals remain as asymptomatic carriers, most infected individuals die of the infection without notice and hence called as a “silent killer” [1].

The prevalence of chronic HBV infection varies greatly in different part of the world. According to World Health Organization (WHO) classification, the world-wide prevalence of chronic HBV infection can range from high (above 8 % as in most resource-limited settings) to low (below 2 % as in most developed settings) [2].

Viral hepatitis is a leading cause of maternal complication and vertical transmission that cause fetal and neonatal hepatitis which can have serious effects on the neonate, leading to impaired mental and physical health later in life [3]. A leading cause of maternal mortality is also said to be the most familiar cause of jaundice in pregnancy [4]. Perinatal transmission of HBV often occurs when the mother presents with acute infection during late pregnancy, in the first postpartum or if the mother is a chronic HBsAg carrier [5].

Hepatitis B virus (HBV) and Human immunodeficiency virus (HIV) infections are posing huge health impact throughout the world and the problem is higher in developing countries, very particularly, in Africa. The two most significant viruses share similar ways of transmission in humans, which accounts for the high frequency of HIV-HBV co-infections [6].

Co-infection with hepatitis B virus and HIV leads to increased morbidity and mortality as compared to independent HIV and HBV infections. In areas where HBV infection is either endemic or intermediate to high, the prevalence rate of HIV/HBV co-infection is recorded as high as 10–20 %. The prevalence rate can be as high as 20–25 % in countries where the viruses are highly endemic [7–9]. The two most significant infectious agents are transmitted by sexual intercourse and positive mothers to fetus or newborns. Studies conducted in Ethiopia have revealed that HBV and HIV are endemic with regional variation [10–12].

In Ethiopia as part of other sub-saharan Africa countries, the prevalence of HIV and liver disease is high and posing a great public health problem. Apart from its significant prevalence, liver disease contributes approximately 12 % of the hospital admissions and 31 % of the mortality in medical wards of Ethiopian Hospitals [13].

The dual burden of HBV and HIV infection in Ethiopia was revealed by many seroepidemiological researches conducted in different segments of the country with regional variation. In a study from a rural hospital in Southern Ethiopia, the seroprevalence was 6.1 % for HBV and 1.8 % for HIV. Co-infection with HIV-1 and HBV was detected in one patient (prevalence: 0.6 %) [14]. According to a hospital-based cross-sectional study conducted in Felege Hiwot Referral Hospital, northwest Ethiopia, the seroprevalence of hepatitis B infections was found to be 4.4 % [15].

A cross-sectional study which was conducted in Addis Ababa to investigate seroprevalence and transmission of Hepatitis B virus among delivering women revealed that 8/265 (3.0 %) of mothers were positive for Hepatitis B Virus surface antigen [16].

Another study from Bahir Dar which has included a total of 318 pregnant women was carried out to determine seroprevalence and risk factors of hepatitis B virus and human immunodeficiency virus infection among pregnant women. Overall, 21/318 (6.6 %) and 12/318 (3.8 %) of the pregnant women were positive for HIV and HBsAg, respectively. Of these, HIV/HBV co-infection rate was 4 (19.0 %) [17].

As part of reducing maternal and child mortality to achieve one of the millennium development goals (MDGs) [18], Ethiopia rolled out childhood immunization against HBV in 2007. The vaccine is delivered in a pentavalent form as part of expanded program on immunization (EPI) of newborns. Despite this effort, newborns are vaccinated without a prior screening of mothers for underlying hepatitis infection. This study aims to assess the current estimate of HBV infection among pregnant mothers attending one of the antenatal care facilities around Addis Ababa and investigate possible factors for HBV infection.

## Results

### General characteristics

The study included a total of two hundred fifteen pregnant women. Table 1 indicates general characteristics of ANC attendant. The median age of ANC attendant was 26 ± 3.94 years. Of the total, 211 (98.1 %) were married who were found to be in monogamous relationship and majority, 164 (76.3 %) were orthodox religion followers. 84 (39.1 %) attended primary school and majority, 129 (60 %), were house wives.

**Table 1:** Socio-demographic characteristics distribution among pregnant women attending Tirunesh Beijing Hospital, 2014, Addis Ababa, Ethiopia

Socio-demographic	Frequency	
Variables	Number (*n*)	Percent (%)
Residence		
Urban	205	95.3
Rural	10	4.7
Age (median = 26 years)		
15–19	9	4.2
20–24	67	31.2
25–29	100	46.5
30–34	31	14.4
35–39	8	3.7
Stage of pregnancy		
First trimester	10	4.7
Second trimester	17	7.9
Third trimester	188	87.4
Educational status		
Illiterate	40	18.6
Primary school	84	39.1
Secondary school	55	25.6
Certificate and diploma	24	11.2
Degree and above	12	5.6
Occupational status		
House wife	129	60
Government organization	19	8.8
Private organization	41	19.1
Merchant	9	4.2
Farmer	9	4.2
Student	3	1.4
Others	5	2.3

### HBV prevalence and analyses of risk factors

The overall seroprevalence for HBsAg among the study population was about 6 % (13/215). There was an overall trend of higher seropositivity for HBV infection among older age groups (between 35 and 39 years) 2 (25 %) compared to the other age groups: 11.1 % among (15–19 years), 4.5 % among (20–24 years), 6 % among (25–29 years), and 3.2 % among (30–34 years). The level of literacy in the majority of the positive cases was low; 8 (9.5 %) were from a primary school level. Of the total, 9 (4.19 %) participants were HIV positive and prevalence of HIV/HBV co-infection was 22.2 % (two HBV positive cases out of nine HIV positive cases).

A multivariate analysis of different variables indicated that those having a history of abortion had a 19 times more chance of positivity for HBsAg than those without [AOR = 19 (CI 2.78–130.367); *p* = 0.003]. Similarly, those with a history of previous surgery had a 9.8 times more chance of positivity for HBsAg compared to their counterparts [AOR = 9.8 (CI 1.392–69.610); *p* = 0.022]. Moreover, the odds of having HBsAg positivity was 7.7 times more likely among those who were with experience of tattoo [OR = 7.7 (CI 1.185–50.28); *p* = 0.033]. Except abortion, surgery, and tattoo, other risk factors included in the study had not showed statistically significant association (*p* > 0.05) with the HBsAg positivity. HBV prevalence and association with risk factors are shown in Table 2.

**Table 2:** HBsAg status and association with potential risk factors among pregnant women at Tirunesh Beijing Hospital, 2014 (*n* = 215)

Risk factors	HBsAg result				
	Positive	Negative	COR (CI)	AOR (CI)	*P* value
Abortion					
Yes	10	35	15.905 (4.162–60.78)	19 (2.78–130.367)	0.003
No	3	167	Ref.	–	
Blood donation					
Yes	5	2	62.5 (10.481–372.702)	20.76 (0.761–566.8)	0.072
No	8	200	Ref.	–	
History of hospitalization					
Yes	8	27	10.370 (3.159–34.040)	0.772 (0.072–8.298)	0.831
No	5	175	Ref.	–	
Surgery					
Yes	11	28	34.179 (7.192–162.422)	9.84 (1.392–69.610)	0.022
No	2	174	Ref.	–	
History of injection					
Yes	12	166	2.6 (0.328–20.656)	1.10 (0.087–14.026)	0.939
No	1	36	Ref.	–	
Tattooing					
Yes	9	44	8.08 (2.375–27.482)	7.720 (1.185–50.28)	0.033
No	4	158	Ref.	–	
HIV status					
Yes	2	7	5.065 (0.939–27.306)	8.201 (0.346–194.423)	0.193
No	11	195	Ref.	–	

## Discussion

The present study will contribute to the understanding of the current burden of HBV among pregnant mothers attending antenatal clinics. In addition, it will significantly contribute to providing insights to the current practice of HBV vaccination in newborns in Ethiopia as newborns are given the vaccine without screening. Therefore, the findings of this study will provide insights for policy makers to implement the routine practice of screening and immunization of pregnant mothers during their antenatal visits.

Screening apparently healthy pregnant women does have a paramount importance from the perspective of particular disease investigation, diagnosis, and implementation evidence-bases medical intervention for chronic HBV infection. Especially, this should be given due emphasis in pregnant women so as to prevent the transmission of HBV to their new born [19].

In agreement with World Health Organization (WHO) grouping, the prevalence of HBV (6 %) was intermediate (2–7 %) [20]. The finding was higher than research report from Jimma (3.7 %), Addis Ababa (3 %), and Bahir Dar (3.8 %), Ethiopia [10, 16, 17]. In comparison with other countries, it was turned out to be higher than a study from Turkey (2.8 %) [21], India (0.9 %) [22], and Libya (1.5 %) [23]. In contrast, it was lower than the study documented in Taiwan (15.5 %) [24] and Gondar in Ethiopia (7.3 %) [12]. These differences might be attributable to socio-demographic characteristics, cultural and behavioral differences for the risk factors of HBV infection, methodological difference, and the obvious natural difference linked with various geographical situations.

HBsAg sero-status was comparable with research reports from Debre Tabor (North West) (5.3 %) [25] and Niger (5.6 %) [26]. The explanation for this could be because we have engaged the same risk groups. In addition, this similarity would be justified by the test method principle similarity employed to investigate HBsAg.

According to previous epidemiological studies, there has been a link between age and the acquisition of HBsAg that indicates, the age of acquiring the infection as one of the major determinant factors for HBsAg positivity [27]. In our study, higher prevalence was observed in the age groups greater than 25 years in agreement with study from Addis Ababa Ethiopia (*p* > 0.05) [14] and China (*p* = 0.001) [28]. Furthermore, a study from Saudi Arabia showed that HBV prevalence is higher among age greater than 25 years compared to those less than 25 years [29].

In Ethiopia, the overall adult HIV prevalence has remained low. The prevalence among adults of age 15–49 in the 2011 EDHS was 1.5 percent. Among regions, HIV prevalence was highest in Gambela (6.5 percent) and in Addis Ababa (5.2 percent) [30]. In this study, the overall HIV prevalence was 4.2 % which was lower than research report from Bahir Dar and Gondar, Ethiopia, respectively [12, 17]. HIV prevalence was higher than the study conducted in rural hospital in Southern Ethiopia (1.8 %) [14] and Mali (0.4 %) [31].

Age-wise HIV prevalence revealed that majority of HIV positive cases were distributed within age group of 25–29 years. In contrary, study from Gonder, Ethiopia [12] and Nigeria (8.4 %) [20] reported higher HIV prevalence in the age group of 21–24 years. But as a matter of fact, aforementioned age categories are highly subjected age group for HIV and sexually transmitted infection which share the same transmission mode like HBV, HCV, and the likes. The most probable explanation could be related with sexual or other risky health behaviors.

The prevalence of HIV/HBV co-infection, accounting 2 (22.2 %), was higher than the study done in Southern Ethiopia (0.6 %) [14] and Nigeria (9.5 %) [20]. In contrast, current HIV/HBV co-infection was comparable with a study conducted in Bahir Dar, Ethiopia (19 %) [17]. Most of the time, appearance of HBV and HIV infection is common among risk groups, because both HBV and HIV share common mode of transmission.

In multi-variate analysis, pregnant women with history of abortion were about 19 times more likely to be positive for HBsAg (AOR = 19; CI = 2.78–130.367); (*p* = 0.003). This variable was significantly associated with HBsAg positivity [32]. Study participants who have had body tattooing on any part of their body was also 7.7 times more likely to be HBsAg positive (AOR = 7.7; CI 1.185–50.28; *p* = 0.033) than their counterparts. Our finding was supported by studies conducted in Mali [33] and Addis Ababa [11], Ethiopia which reported that body tattooing on any part shown to be significant predictor of HBV prevalence. Moreover, undergoing surgical procedure was significantly associated with and important predictors of hepatitis B infection (AOR = 9.8; CI = 1.392–69.610; *p* = 0.022) [34].

The sero-markers that we have used to detect HBV infection were only HBSAg and it was not possible to investigate the transmission rate. Earlier studies in Ethiopia, on the other hand, showed a minimal risk of perinatal transmission of HBV [35]. However, researches conducted elsewhere estimated that transmission rates can be as high as 12, 25, and 70–90 %, respectively, in HBeAg-negative/anti-HBe-positive mothers, HBeAg-negative/anti-HBe-negative mothers, and HBeAg-positive/anti-HBe-negative mothers [36].

According to research findings, a perinatal transmission was found to be associated with HBeAg positivity and viral load. It was noted that the risk of transmission was significantly higher from HBeAg-positive mothers compared with HBeAg-negative mothers, and from mothers with a very high viral load compared with mothers with high or low viral loads. Perinatal transmission was not seen in babies born to mothers with HBV DNA levels <108 copies/mL or mothers who were negative for HBeAg [37].

By considering that intrauterine transmission is the major way of HBV transmission from positive mothers to their fetus/newborn, particularly in HBV endemic countries, all pregnant women should be screened for hepatitis B virus and those found to be positive need to be given HBIG prophylaxis [14]. Immunizing newborns with the hepatitis B vaccine should be the highest priority in highly endemic areas where the contribution of perinatal transmission to the overall disease burden is greatest. However, even in countries with a relatively low prevalence of chronic HBV infection, implementation of a birth dose of hepatitis B results in an additional 10–20 % reduction in HBV mortality [38, 39].

Taking into account the impact of viral hepatitis due to the leading route of mother-to-child transmission, designing and implementing intervention strategies to reduce the vertical transmission of HBV and HCV have paramount importance. Administration of at-birth prophylaxis of newborns of HBV-infected mothers with specific immunoglobulin and vaccine plus administration of antivirals (tenofovir or telbivudine) in the third trimester of pregnancy (in case of high maternal viral load) greatly reduce the risk of transmission [40].

While treating, continuing, and stopping HBV therapy, all decisions required about the risks and benefits for both the mother and fetus. In addition, the trimester of the pregnancy and the stage of the mother’s liver disease are important factors. Treatment in the third trimester may be considered to aid in the prevention of perinatal transmission, which appears to be most pronounced in mothers with high viral loads [41].

Maternal-infantile transmission is a major way of transmission route for HBV and HIV; strategies targeted at disrupting this pathway would greatly diminish the number of new infections and would mitigate the suffering imposed by the disease on the individuals, families, society and the country at large society. As a result, we recommend that studies conducted in the future need to consider HBeAg seromarker in order to detect intrauterine transmission rate.

Prevention of HBV infection, which can be done through vaccination, is a crucial measure so as to minimize the world-wide occurrence of HBV infection. Vaccine-mediated intervention is preferred over the other way of healthcare interventions for its being economically advantageous. As per the year 1991 WHO recommendation, all countries were urged to introduce a health policy of universal hepatitis B vaccination to prevent and control HBV infection and its long-term sequelae on a global scale [42, 43].

In Ethiopia, EPI was launched at nation-wide scale in 1980 with the aim of providing immunization services to all children under the age of 2 years [44]. In the light of the WHO recommendations and the epidemiological and medical picture, Ethiopia is a candidate for the introduction of HBV vaccination as part of the National EPI program. Despite a number of studies conducted to assess the impact of HBV infection, Ethiopia rolled out infant immunization against hepatitis in 2007. Currently, the vaccine is provided as part of expanded program on immunization (EPI), despite no routine screening of expecting mothers.

The prevalence of HBV infection in the pregnant women, though lower than other reports conducted elsewhere, is still important. For the purpose of the study, only HBsAg was determined which tells about active infection rather than total infection rate. In contrary, investigation of seromarkers including anit-HBC and HBC antigen would have great potential to trace the overall distribution of HBV infection and indicators of the total infection rate in a certain population. These can be considered as the major limitation of this study. Moreover, unfortunately, it was difficult to follow-up these women till delivery to establish vertical transmission and its associated factors.

## Conclusions

The present study showed an intermediate prevalence of HBV infection among pregnant women according to World Health Organization’s classification. A 6 % overall prevalence of HBV infection in our study setting among pregnant mothers attending antenatal care facility indicates the need for timely intervention strategies to alleviate the burden of HBV infection in the nearby community.

Although earlier studies indicated a minimal role for perinatal and vertical transmission of HBV infection, the impact of health education cannot be neglected, particularly with the observed risk factors such as body tattooing, abortion, and previous history of surgery as a direct inflict of existing health facilities.

Furthermore, the observed prevalence might also warrant the introduction of routine screening of hepatitis in all pregnant women and those in the reproductive age group by large. This would particularly be important, considering the current practices of infant immunization with HBV that is provided without prior screening of expecting mothers. However, since there is no treatment guidelines neither confirmatory tests available in most health facilities in Ethiopia at the moment, a wider scale study with a larger cohort of population in different health facilities is warranted.

## Methods

### Study setting and population

This cross-sectional study was conducted in one of the general hospitals, Tirunesh Beijing Hospital, located at the periphery of Addis Ababa, Ethiopia, between July and October 2014. A total of 215 pregnant women whose age was greater than 18 years, attending antenatal clinic (ANC) at the Tirunesh Beijing Hospital, were recruited consecutively. All socio-demographic information were collected from all participants using a standard questionnaire that was pretested. All participants gave written informed consent before enrollment to the study and test results were communicated for proper management and care of the study participants.

### Sample collection and detection of antibody for Hepatitis surface antigen (HBsAg)

Five ml of venous blood was collected aseptically and centrifuged to separate the serum. Serum samples were then stored at −20 °C until assayed for antibodies against hepatitis surface antigen (HBsAg**)** using one-step HBsAg test strip (Linear Chemicals, Joaquim Costa, Barcelona, Spain). In addition to HBsAg, samples were screened for HIV antibody using commercially available rapid test kits in accordance with the national HIV test algorithm. To check for the reliability of the tests, we strictly followed the manufacturers’ instructions; both positive and negative controls were run alongside of the tests.

### Statistical analysis

Data were checked for completeness and entered into EPI-INFO Version 3.5.1. And then statistical package SPSS software for windows, ver. 20 was used for all analyses. Computed descriptive statistics were presented using texts and tables. Potential risk factors association with occurrence HBV infection was identified using bivariate and multivariate analysis. Multivariable logistic regression model was used to identify the relative importance of each predictor to the dependent variable by controlling for the effect of other variables. Exposure variables having statistically significant association with HBV infection after controlling the effect of other variables were considered as predictors of the outcome variables. Strength of association was measured using odds ratio (OR) with 95 % confidence interval (CI). Differences were considered significant if the *p* value was <0.05.

### Ethical considerations

Ethical clearance and approval was obtained from Armauer Hansen Research Institute (AHRI)—All Africa Leprosy and Tuberculosis Rehabilitation and Training Center (ALERT) Ethics Review Committee (AHRI/ALERT ERC) (*Project Reg. No: PO42/14*). The study protocol was carefully explained to the participants and participation was fully voluntary. Written informed consent was obtained from all participants. All procedures were done according to the standard with a minimum risks. Study results were returned to respective care givers and incorporated into their care.

## Abbreviations

AHRI: Armauer Hansen Research Institute
ALERT: All Africa Leprosy and Tuberculosis Rehabilitation and Training Center
ANC: antenatal care
DNA: deoxyribonucleic acid
ELISA: enzyme-linked immunosorbent assay
HBeAg: hepatitis B e antigen
HBsAg: hepatitis B surface antigen
HBV: hepatitis B virus
HCV: hepatitis C virus
HIV: human immunodeficiency virus
STIs: sexually transmitted infections
WHO: World Health Organization
